# Association between Vitamin D Status and Coronary Heart Disease among Adults in Saudi Arabia: A Case-Control Study

**DOI:** 10.3390/healthcare4040077

**Published:** 2016-10-17

**Authors:** Najlaa M. Aljefree, Patricia Lee, Jamal M. Alsaqqaf, Faruk Ahmed

**Affiliations:** 1 Public Health, School of Medicine and Menzies Health Institute Queensland, Gold Coast Campus, Griffith University, Southport, QLD 4222, Australia; patricia.lee@griffith.edu.au (P.L.); f.ahmed@griffith.edu.au (F.A.); 2 Department of Cardiology, King Abdulla Medical City (KAMC), Makkah 21955, Saudi Arabia; drjamal70@gmail.com

**Keywords:** cardiovascular disease, vitamin D deficiency, diabetes, obesity, Saudi Arabia, Middle East

## Abstract

Recent evidence has pointed out an association between vitamin D deficiency and coronary heart disease (CHD). Due to the growing epidemic of CHD and vitamin D deficiency in Saudi Arabia, exploring the role of vitamin D in the prevention of CHD is crucial. The aim of this study was to examine the association between vitamin D status and CHD in Saudi Arabian adults. This case-control study included 130 CHD cases and 195 age-sex matched controls. Study subjects were recruited from three hospitals in the western region of Saudi Arabia. Study participants were interviewed face-to-face to collect data on their socio-demographic characteristics and family history of CHD. Fasting blood samples were collected, and serum levels of vitamin D, glucose, and total cholesterol were measured. Body weight, height, and blood pressure measurements were also recorded. Severe vitamin D deficiency (25(OH)D < 10 ng/mL) was much more prevalent in CHD cases than in controls (46% and 3%, respectively). The results of multivariate logistic regression showed that vitamin D deficiency (25(OH)D < 20 ng/mL) was associated with CHD, with an odds ratio of 6.5 (95% CI: 2.7–15, *p* < 0.001). The current study revealed that vitamin D deficiency is independently associated with CHD, suggesting an important predictor of CHD among Saudi adults.

## 1. Introduction

Cardiovascular disease (CVD) is one of the most common causes of death and disability globally. The Gulf region, including Saudi Arabia, is facing a massive burden of CVD and associated risk factors [1]. Data from hospitals have indicated that CVDs are the leading cause of hospital admissions in Saudi Arabia, and coronary heart disease (CHD) is the third most prominent cause of hospital-based mortality in the country, after traffic accidents and senility [2]. In Saudi Arabia, CHD was found to be prevalent among 5.5% of the population [3]. Furthermore, national-level studies have reported a significant burden of CHD risk factors among the Saudi population, including obesity, diabetes, and hypercholesterolemia [4,5,6,7]. However, besides known relationships between traditional risk factors (such as obesity, diabetes, and hypertension) and CHD, other important factors, including poor dietary habits and deficiency of micronutrients such as vitamin D, have recently been found to be associated with the burden of CHD and other chronic diseases [8].

Evidence to date suggest that vitamin D deficiency may negatively affect the cardiovascular system by activating the renin-angiotensin-aldosterone system, which leads to the development of hypertension and left ventricular hypertrophy [9,10]. In addition, low levels of vitamin D can result in an increase in parathyroid hormone (PTH), which causes an increase in blood pressure as well as myocardial contractility. This in turn may lead to hypertrophy and fibrosis of the left ventricle and vascular medial smooth muscles [9,10]. Large epidemiological studies such as the National Health and Nutritional Examination Surveys (NHANES) and the Framingham Offspring cohort have shown that low levels of vitamin D were independently associated with a higher risk of myocardial infarction, heart failure, and stroke [11,12]. Furthermore, a meta-analysis of 24 studies reported an inverse association between vitamin D deficiency and risk of CVD [13]. Likewise, a recent meta-analysis of eight prospective cohort studies showed that serum levels of vitamin D in the lowest quintile were significantly associated with increased all-cause mortality including cardiovascular mortality [14]. However, the previous studies included in the meta-analysis were largely from European countries and the United States; hence, they may not be culturally or ethnically appropriate for the Saudi population. In the Middle Eastern countries, few studies have focused on the association between vitamin D status and the risk of CVD [15,16]. To our knowledge, no previous studies among Saudi adults have been carried out that focused on the relationship between vitamin D status and the risk of CHD. In Saudi Arabia, vitamin D deficiency is highly prevalent, even though there is plentiful sunlight throughout the year [17,18,19,20,21,22,23]. Several studies have reported low levels of vitamin D ranging from 8.4 to 11.6 ng/mL and from 8 to 16.6 ng/mL in males and females, respectively [17,18,22,23,24]. Because both CHD and vitamin D deficiency are significant problems in the Saudi population, there is a need to examine whether vitamin D deficiency is independently associated with CHD in this population and thereby generate evidence for developing appropriate interventions. Therefore, the present study was designed to examine the association between vitamin D status and CHD among adults in Saudi Arabia.

## 2. Materials and Methods

### 2.1. Study Design and Population

A case-control study design was applied to examine the association between vitamin D status and CHD among adults living in the two largest cities in Saudi Arabia, Jeddah and Makkah, which are both located in the Western region of the country. Study participants were recruited from three hospitals; King Abdullah Medical City (KAMC) and Tunsi private hospital in Makkah and King Abdul Aziz University Hospital (KAU) in Jeddah. Data collection was conducted in the summer between May and October 2015, when the average temperature reached 37 °C and the average daily sunlight was 9 hours in coastal cities such as Jeddah and Makkah. The sample size consisted of 130 CHD cases and 195 controls with a ratio of 1:1.5. The study protocol was approved by the Griffith University Human Research Ethics Committee (GU Ref No: MED/59/14/HREC), Research Ethics Committee in KAU (Reference No ll8-15), and Institutional Review Board in KAMC (IRB No: 15-194).

**Figure 1:**
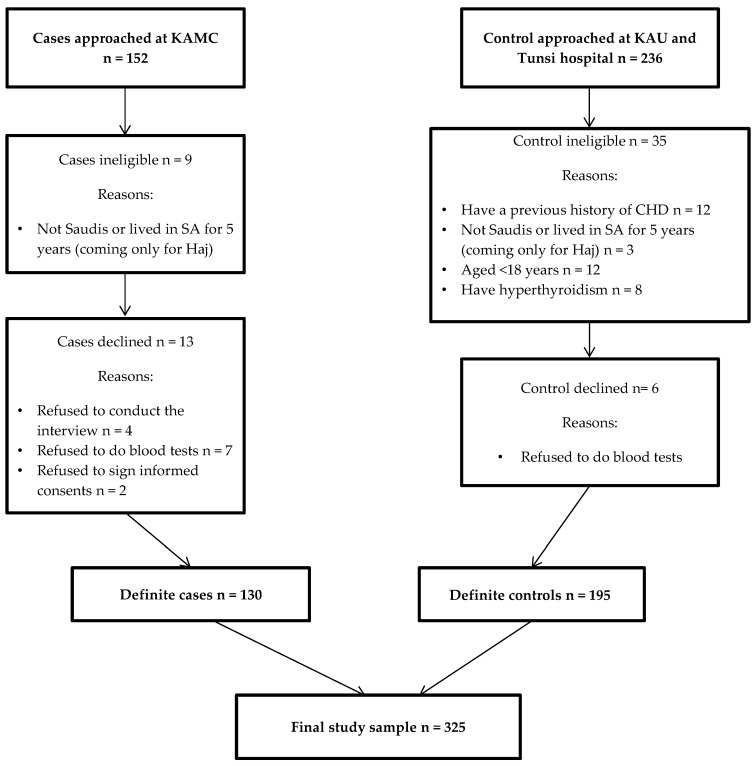
The subject’s recruitment process and reason for refusal to participate in the study.

All CHD cases (n = 130) were recruited from KAMC hospital in Makkah, which provided a good site for recruiting potential subjects for the current study because it has a large cardiac surgical facility and because a large majority of the cardiovascular patients in this hospital have been referred from different areas in the western region of the Kingdom. Cases were recruited from subjects who were admitted to KAMC hospital during the research period and were either first incident with an acute event or have been diagnosed earlier with clinical artery disease, myocardial infarction, or chronic stable angina and met the selection criteria. Controls were recruited from two hospitals, KAU in Jeddah (42 participants) and Tunsi private hospital in Makkah (153 participants). Controls were matched in age (within 0–5 years) and gender and had no history of CVD. They were recruited from ophthalmology clinics in KAU hospital and from family medicine clinics and nose and throat (ENT) clinics in Tunsi hospital. All potential study subjects (152 cases and 236 controls) in the hospitals were approached to participate in the study during the time of data collection. Nine cases and 35 controls were excluded because they did not meet the eligibility criteria. Written informed consent was obtained from eligible subjects before their participation in the study. Of the eligible study subjects, 13 cases and 6 controls refused to participate in the study. Figure 1 shows the subjects’ recruitment process and reasons for refusal to participate in the study.

### 2.2. Inclusion and Exclusion Criteria

Inclusion criteria for both cases and controls included being a native Saudi or resident in Saudi Arabia for at least five years and being an adult of 18 years or older. Exclusion criteria included having a medical condition that could affect vitamin D metabolism, including metabolic bone disorders such as osteoporosis. Subjects were also excluded if they had liver disease, kidney disease, hyperparathyroidism, granulomatous disease, tuberculosis, lymphomas or hyperthyroidism. Furthermore, subjects with malabsorption resulting from celiac disease, Crohn's disease, and bypass surgery were also excluded [25].

### 2.3. Data Collection

Face-to-face interviews were conducted with all participants using a structured questionnaire. Data were collected on participants’ sociodemographic characteristics, such as age, gender, marital status, education level, nationality, and monthly income. In addition, information was collected regarding family history of CVD and behavioral risk factors, for instance, cigarette smoking, water-pipe smoking, and levels of physical activity. Subjects who smoked at least one cigarette per day were considered current smokers. Subjects who had never smoked were considered non-smokers. A previous smoker was a person who had previously smoked but had quit [26]. Water-pipe smokers were subjects who smoked at least one water-pipe per week at the time of the interview [27]. Physical activity was self-reported and classified as moderate activity, such as jogging, walking, or swimming; vigorous activity that causes sweating or hard breathing, such as heavy lifting, aerobics, or fast bicycling; and sedentary, such as staying at home most of the time or doing a little walking outside [26]. The participants were also asked to report the use of dietary supplements, including both dose and duration, for vitamin D and calcium supplements with vitamin D. Similarly, the participants also asked to report sun exposure during weekdays and weekend (times spent outdoor) and the use of sunscreen. Measurement of height (centimeters) and weight (kilograms) in light clothing were taken using a standard scale after the participants were interviewed. Then, body mass index (BMI) was calculated by dividing the weight in kilograms by height in meters squared. Overweight and obesity were defined according to the World Health Organization (WHO) definition. The subjects were considered overweight when BMI was 25.0–29.9 kg/m², and the subjects were defined as obese when BMI was ≥30 kg/m² [28]. The participants were asked to sit for 5 minutes before their blood pressure was measured using standard equipment. Hypertension was defined according to the WHO criteria as a blood pressure ≥140 mmHg for systolic blood pressure (SBP) and/or ≥90 mmHg for diastolic blood pressure (DBP) [28].

### 2.4. Biochemical Measurements

A hematological technician collected 10 milliliters of venous blood from each case and control (325 subjects), using a disposable syringe to assess their serum levels of 25(OH)D, fasting glucose, and total cholesterol. The blood samples were centrifuged at 2000 rpm for 15 minutes, and then serum was separated. All serum samples were kept frozen at −80 °C until further lab analysis. Serum levels of 25(OH)D were measured by chemiluminescence microparticle immunoassay (CMIA) on the Architect system (Abbott) (Wiesbaden, Germany). The intra- and inter-assay coefficients of variation (CVs) were 2.7% and 4.6%, respectively. Fasting glucose and total cholesterol were measured using biochemical analyzer (Thermo Fisher Scientific, Espoo, Finland). The laboratories are located at the same hospitals where the study was undertaken and are certified by the Saudi Ministry of Health. Vitamin D deficiency and insufficiency were defined as serum concentrations of 25(OH)D < 10 ng/mL, and 10 to <19.9 ng/mL, respectively. A serum concentration of 25(OH)D ≥ 20 ng/mL was considered to be an adequate vitamin D level [29]. In the current study, vitamin D deficiency and insufficiency were combined for analysis purposes due to the small sample size; therefore, vitamin D deficiency was defined as having serum concentration of 25(OH)D < 20 ng/mL. Diabetes was defined according to the WHO standard of diagnosis of glucose intolerance when fasting plasma glucose (FPG) was ≥126 mg/dL [28]. High total cholesterol (HC) was defined according to the Adult Treatment Panel III (ATP III) guidelines as HC ≥ 240 mg/dL [28].

### 2.5. Statistical Analysis

Statistical analyses were performed using Statistical Package for Social Science (SPSS) version 22 (IBM SPSS Software, Chicago, IL, USA). A chi-square test was used to assess the association between each independent variable (including vitamin D status) and the outcome status (CHD). For the purpose of regression analyses, the distribution of SBP, DBP, fasting glucose, and total cholesterol were divided onto equal thirds (tertiles). Multivariate logistic regression models were conducted to examine the relationship between vitamin D status and CHD. Vitamin D status was categorized into two groups; vitamin D deficiency was defined as serum 25(OH)D < 20 ng/mL, where vitamin D deficiency and insufficiency were combined together to increase the statistical precision due to the small sample size, and adequate vitamin D status was defined as serum 25(OH)D ≥ 20 ng/mL. The multivariate logistic regression analysis was carried out using different models. The first model was a crude model with no adjustment for confounders. The second model was created by including only socio-demographic variables, such as age, gender, education, employment, citizenship, place of residence, marital status and family monthly income. The final model was created by adding other potential confounders such as BMI, fasting blood glucose, total cholesterol, smoking, exercise, use of vitamin D supplements, use of calcium supplements with vitamin D, time spends for sun exposure and the use of sunscreen. As there were very few subjects in either group of the study sample who smoked a water-pipe, we combined cigarette smoking and water-pipe smoking for the logistic regression. Likewise, due to the low number of subjects in either group who practiced vigorous exercise, moderate and vigorous exercise was combined for the logistic regression. *p < 0.05* was considered statistically significant.

## 3. Results

The socio-demographic characteristics of the CHD cases and controls are shown in Table 1. Almost 65% of cases had first incident with an acute event at the time of data collection and 35% of cases were previously diagnosed with CHD. As expected, there were no significant differences in the distribution of age and gender categories between case and control groups. Eighty-one percent of the cases and 63% of the controls were Saudis. The majority of the cases and controls were married (70% and 72%, respectively) and had low to medium family monthly income (72% and 69%, respectively). Relatively more participants in the control group were highly educated (*p* < 0.001) and doing a paid job (*p* < 0.001) than the participants in CHD cases. Nearly 85% of the participants in the control group were non-smokers, and 59% of participants with CHD were non-smokers (*p* < 0.001). However, practicing moderate exercise (*p* = 0.007) was more common in CHD cases than in controls (Table 1).

The clinical characteristics of the CHD cases and controls are shown in Table 2. Vitamin D deficiency (*p* < 0.001) was significantly higher in CHD cases than in the controls; 46% of the participants in the CHD group were vitamin D deficient (serum 25(OH)D < 10 ng/mL), whereas the rate of deficiency was only 3% in the control group. On the other hand, 61% of the controls had adequate vitamin D levels (serum 25(OH)D ≥ 20 ng/mL) compared to 24% of the CHD cases (Table 2). A significantly higher proportion of the participants in the CHD cases were obese (44% and 22%, respectively) and had higher fasting blood glucose levels (FPG ≥ 126 mg/dL) (35% and 14%, respectively) than those in the control group (*p* < 0.001). A relatively higher proportion of participants in the control group had higher total serum cholesterol levels (≥240 mg/dL) (13% and 5%, respectively) than in the CHD group (*p* < 0.001). There was no significant difference in the distribution of SBP levels between the two groups (Table 2).

The results of the multivariate logistic regression are shown in Table 3. Vitamin D deficiency (serum 25(OH)D < 20 ng/mL) was significantly associated with increased odds of CHD (*p* < 0.001). After adjustment for age, gender, education, employment, citizenship, place of residence, marital status, family income, BMI, blood glucose, total cholesterol, smoking, exercise, and use of vitamin D supplements, calcium supplements with vitamin D, sun exposure, and the use of sunscreen, subjects with vitamin D deficiency (serum 25(OH)D < 20 ng/mL) were 6.5 times more likely to suffer from CHD compared to those with adequate vitamin D levels (serum 25(OH)D ≥ 20 ng/mL) (OR: 6.5, 95% CI: 2.7–15, *p* < 0.001) (Table 3). 

## 4. Discussion

The findings of the present study revealed that the subjects with vitamin D deficiency, when defined as serum 25(OH)D < 20 ng/mL, were 6.5 times more likely to suffer from CHD than the subjects with adequate vitamin D status (serum 25(OH)D ≥ 20 ng/mL). Several studies conducted in developed countries have also demonstrated similar results [30,31]. For example, an inverse association between vitamin D deficiency and myocardial infarction (MI) was reported among adults in New Zealand [32]. In the United States, an NHANES study stated that the participants with vitamin D deficiency had a higher prevalence of angina and MI compared to that in the participants with adequate levels of vitamin D (OR: 1.20 (95% CI: 1.01, 1.36) [33]. In a Gulf country, Qatar, a study indicated that males with vitamin D deficiency had a three times higher risk of MI than males with an adequate vitamin D levels [16]. More recently, a study among an Indian population showed 4.5 times higher risk of MI among subjects with vitamin D deficiency (<10 ng/mL) [34]. It is important to note that different studies across the globe have used different criteria for defining vitamin D deficiency, and the reason for this is that the accurate cut-off value for defining vitamin D deficiency remains controversial. There is disagreement surrounding serum PTH, which is inversely associated with low levels of vitamin D. Some studies have suggested that the production of PTH escalates when serum levels of 25(OH)D are less than 10 ng/mL, which leads to bone loss and fractures [35]. However, other studies have indicated that levels of serum 25(OH)D ranging from 18 ng/mL to 30 ng/mL lead to increased PTH levels and cause bone loss [29]. Nevertheless, regardless of the definition used to assess the association between vitamin D status and CHD, a large majority of the studies showed an inverse association similar to what was found in the present study.

The protective role of vitamin D against CHD could be explained by the wide distribution of vitamin D receptors (VDRs) in the vascular walls, which plays a crucial role in cardiac physiology [36]. Animal studies have shown a direct effect of the absence of VDRs on cardiac function. These studies, which genetically modified the animals to have no vitamin D receptors or no 1, 25 (OH)2 D, indicated that they developed left ventricular hypertrophy and heart failure [36]. The results of animal studies were corroborated by the findings observed in patients with end-stage renal disease (ESRD) [37]. Human ESRD studies provided one of the first pieces of evidence that supported the role of vitamin D deficiency in the development of CHD. Due to damage in the kidneys, ESRD patients failed to convert 25 (OH) D into 1, 25 (OH)2 D, which in turns leads to increased levels of PTH. The high level of PTH causes elevated blood pressure and cardiac contractility, which contributes to myocardial dysfunction, arterial hypertension, and heart failure [9,36,37].

The current research revealed 61% prevalence of adequate vitamin D levels (≥20 ng/mL) among the healthy subjects (controls), which appears to be consistent with previous studies within Saudi Arabia. Naeem et al. showed that the mean vitamin D levels were 32 ng/mL and 23 ng/mL among Saudi males and females, respectively [20]. Likewise, Alsuwaida et al. stated that 50% of study participants had adequate vitamin D levels ≥ 30 ng/mL [38]. A national survey conducted in Saudi Arabia in 2013 among Saudis from both genders aged 15 years and above reported that vitamin D deficiency (<28 ng/mL) was prevalent among 40.6% of males and 62.6% of females [39].

This study has several strengths. It was not restricted to a specific gender, as it examined both males and females. Furthermore, it has demonstrated the association between vitamin D deficiency and CHD after controlling for a wide range of socio-demographic factors and behavioral confounding factors such as using vitamin D supplements, time spent outdoor for sun exposure, and the use of sunscreen. In addition, data related to CHD risk factors were measured at the same hospitals but not self-reported, which added more value to the results. Finally, this study indicated an independent association of vitamin D deficiency with CHD, which has never been explored before in Saudi Arabia.

However, this research has a number of limitations. First, the research used a case-control design, which can only draw inferences about the association between the exposure and outcome variables but not deduce the casual relationship. Second, the measurements of serum vitamin D concentration were not done before the diagnosis of cases. However, a case-control study provides stronger evidence than a cross-sectional design. Third, recall bias might be an issue in this study, as both cases and controls were asked to remember information, including their smoking history. Fourth, case and control subjects were recruited from different hospitals, which might have introduced some selection bias. It is noteworthy that the data collection was conducted during Haj season (Muslim pilgrimage), and the KAMC hospital closed the outpatient’s clinics to the public except for pilgrims in order to deliver health services to them. Thus, it was not possible to recruit control subjects from KAMC hospital in Makkah, from where the cases were recruited. Consequently, the control subjects were recruited from other hospitals located in Makkah and Jeddah. However, these hospitals were situated within a distance of only 70 km, and both were in the western region of the Kingdom. Further, any differences in socio-demographic characteristics between cases and controls were taken into account in the final regression model in order to minimize the effect of selection bias. Fifth, a single measurement of vitamin D status was another limitation as 25 (OH)D has a half-life of up to three weeks and it only reflects the current status [10]; thus, multiple measurements would have been the best reflection of the average of vitamin D status. Finally, the duration of time since diagnosis with CHD for cases who were previously diagnosed was not collected; however, only one-third of cases were diagnosed with CHD earlier.

## 5. Conclusions

The current study revealed an inverse association between vitamin D status and CHD among adults in Saudi Arabia after adjusting for potential confounding factors. Findings of the present study have important implications for future strategies for CHD prevention by improving vitamin D status among adults in Saudi Arabia.

## Figures and Tables

**Table 1:** Socio-Demographic, Family History of CVD, and Lifestyle Behaviors Characteristics of Case and Control Subjects.

Variable	Cases (n = 130)%	Control (n = 195)%	*p*-Value
Age (years)			
<49	25	30	
≥49	75	70	0.340
Gender			
Male	63	63	
Female	37	37	>0.05
Marital status			
Single	5	17	
Married	70	72	<0.001
Divorced	25	11	
Citizenship			
Saudis	81	63	
Non-Saudis	19	37	<0.001
Place of residence			
Rural	12	1	
Urban	86	98	
Semi-rural	2	1	<0.001
Education			
Up to primary levels	51	14	
High School and bachelor or diploma degree	25	35	<0.001
Master or PhD degree	24	51	
Employment			
Employed (Full time, Part time, self-employed)	32	82	
Unemployed (Student, Retired, Housewife)	68	18	<0.001
Family income (SR */monthly)			
<5000	72	69	
5000–15000	10	19	
15000 ≥ 25000	18	12	0.036
Smoke cigarettes			
Current <20 cigarettes/day	15	10	
Previous smoker	26	5	
Non-smoker	59	85	<0.001
Water pipe smoker			
Yes	3	10	
No	97	90	0.022
Moderate exercise			
Never and rarely	34	44	
1–2 times/week	17	24	
More than 3–4 times/week	49	32	0.007
Vigorous exercise			
Never and rarely	98	96	
1–2 times/week	1	0	
More than 3–4 times/week	1	4	0.259
Family history of CVD			
Yes	41	42	
No	59	58	0.890

*p*-Value based on X² -test; * Saudi Riyal (1SR = 0.37 AUD).

**Table 2:** Clinical characteristics of case and control subjects.

Variable	Cases (n = 130)%	Control (n = 195)%	*p*-Value
BMI			
Normal weight < 25 kg/m²	31	33	
Overweight 25–29.9 kg/m²	25	45	<0.001
Obese ≥ 30 kg/m²	44	22	
SBP			
<112.58 mmHg	35	31	
112.59–128.42 mmHg	29	38	0.031
≥128.43 mmHg	36	31	
DBP			
<69.58 mmHg	56	17	
69.59–79 mmHg	23	43	<0.001
≥79.1 mmHg	21	40	
Fasting glucose (FPG)			
<93 mg/dL	21	42	
93.1–112.42 mg/dL	28	37	<0.001
≥112.43 mg/dL	51	21	
Total Cholesterol			
<154 mg/dL	56	20	
154.1–193 mg/dL	28	37	<0.001
≥193.1 mg/dL	16	43	
Vitamin D			
Adequate ≥ 20 ng/mL	24	61	
Insufficient 10 to < 19.9 ng/mL	30	36	<0.001
Deficiency < 10 ng/mL	46	3	

*p*-Value based on X² -test; BMI, body mass index; SBP, systolic blood pressure; DBP, diastolic blood pressure; FPG, fasting plasma glucose.

**Table 3:** Odd ratios (95% confidence interval) for CHD among subjects with Vitamin D deficiency in adults in Saudi Arabia.

Vitamin D status	Crude OR ^1^ (95% CI)	Adjusted OR ^2^ (95% CI)	Adjusted OR ^3^ (95% CI)
Adequate (≥20 ng/mL)	1.00 (referent)	1.00 (referent)	1.00 (referent)
Deficient (<20 ng/mL) *	5 (3.04–8.20) <0.001	7.8 (3.79–16.3) <0.001	6.5 (2.7–15) <0.001

* Defined as combined vitamin D insufficiency and deficiency; ^1^ Multivariate Logistic Regression model with no adjustment; ^2^ Multivariate Logistic Regression model after adjustment for age, gender, education, employment, citizenship, place of residence, marital status, and family income; ^3^ The final model after additional adjustment for smoking, exercise, BMI, blood glucose, total cholesterol, vitamin D supplements, calcium supplements with vitamin D, sun exposure, and the use of sunscreen.
